# Effects of functional feeds on the lipid composition, transcriptomic responses and pathology in heart of Atlantic salmon (*Salmo salar* L.) before and after experimental challenge with Piscine Myocarditis Virus (PMCV)

**DOI:** 10.1186/1471-2164-15-462

**Published:** 2014-06-11

**Authors:** Laura Martinez-Rubio, Øystein Evensen, Aleksei Krasnov, Sven Martin Jørgensen, Simon Wadsworth, Kari Ruohonen, Jose LG Vecino, Douglas R Tocher

**Affiliations:** Institute of Aquaculture, School of Natural Sciences, University of Stirling, Stirling, FK9 4LA Scotland, UK; Faculty of Veterinary Medicine, Norwegian Life Science University, PO Box 8146, Dep, Oslo, N-0033 Norway; Nofima AS, PO Box 210, Ås N-1431 Kragujevac, Norway; EWOS Innovation AS, Dirdal, N-4335 Norway

## Abstract

**Background:**

Cardiomyopathy syndrome (CMS) is a severe cardiac disease of Atlantic salmon (*Salmo salar*) recently associated with a double-stranded RNA virus, Piscine Myocarditis Virus (PMCV). The disease has been diagnosed in 75-85 farms in Norway each year over the last decade resulting in annual economic losses estimated at up to €9 million. Recently, we demonstrated that functional feeds led to a milder inflammatory response and reduced severity of heart lesions in salmon experimentally infected with Atlantic salmon reovirus, the causal agent of heart and skeletal muscle inflammation (HSMI). In the present study we employed a similar strategy to investigate the effects of functional feeds, with reduced lipid content and increased eicosapentaenoic acid levels, in controlling CMS in salmon after experimental infection with PMCV.

**Results:**

Hepatic steatosis associated with CMS was significantly reduced over the time course of the infection in fish fed the functional feeds. Significant differences in immune and inflammatory responses and pathology in heart tissue were found in fish fed the different dietary treatments over the course of the infection. Specifically, fish fed the functional feeds showed a milder and delayed inflammatory response and, consequently, less severity of heart lesions at earlier and later stages after infection with PMCV. Decreasing levels of phosphatidylinositol in cell membranes combined with the increased expression of genes related with T-cell signalling pathways revealed new interactions between dietary lipid composition and the immune response in fish during viral infection. Dietary histidine supplementation did not significantly affect immune responses or levels of heart lesions.

**Conclusions:**

Combined with the previous findings on HSMI, the results of the present study highlight the potential role of clinical nutrition in controlling inflammatory diseases in Atlantic salmon. In particular, dietary lipid content and fatty acid composition may have important immune-modulatory effects in Atlantic salmon that could be potentially beneficial in fish balancing the immune and tissue responses to viral infections.

**Electronic supplementary material:**

The online version of this article (doi:10.1186/1471-2164-15-462) contains supplementary material, which is available to authorized users.

## Background

Cardiomyopathy syndrome (CMS) is a severe cardiac disease of Atlantic salmon (*Salmo salar*) recently associated with a double-stranded RNA virus termed piscine myocarditis virus (PMCV) [1]. The disease was first described and diagnosed in Norway in 1985 [2], and has since also been diagnosed in the Faroe Islands [3], Scotland [4] and, possibly, Canada [5]. In 2012, the Norwegian Veterinary Institute diagnosed the presence of CMS at 89 salmon farming sites located in the mid-coast of Norway [6]. Early stages of the disease have been reported in adult Atlantic salmon close to harvest, around 14 to 18 months after transfer to seawater [7], and so the economic impact can be considerable [8].

Histopathologically, CMS is characterized by severe inflammation and necrosis of the spongy myocardium of the atrium and ventricle [9], but liver may also be affected due to the circulatory disturbance associated with the heart lesions. Mortality is usually moderate although morbidity can be very high with the associated chronic inflammatory response lasting for several months. The pathogenesis of experimentally induced CMS was previously assessed by transcriptomic profiling using oligonucleotide microarrays in association with pathology [10, 11]. In these studies, cardiac pathology and viral load were correlated, with an up regulation of T cell response genes. It appeared that the cellular effector response mediated by CD8+ T cells contributed to successful clearance of the virus infection, although it was also correlated with an up regulation of apoptotic genes, which could contribute to tissue damage.

The lack of commercial vaccines to CMS currently makes the use of alternative therapies crucial. Factors that modulate the inflammatory process might be key to mitigate the clinical symptoms and improve the performance of affected fish. The concept of clinical nutrition is well known in humans [12], and is becoming of increasing interest in aquaculture. Functional feeds are defined as high-quality feeds that, beyond their nutritional composition, are formulated with health promoting features that could be beneficial in supporting disease resistance and mitigation of clinical disease symptoms. Thus a clinical nutrition approach using functional feeds potentially enables a shift away from chemotherapeutic and antibiotic treatments, lowering the costs of disease treatment and management [13]. There are several studies in fish linking nutrition and immunology, recently reviewed by Kiron (14). The inclusion in aquaculture feeds of additives such as prebiotics, probiotics, immunostimulants, vitamins and nucleotides, is reported to increase growth and feed conversion efficiency, as well as having positive effects on the immune system and protection against bacterial infections [13]. Moreover, macronutrients like proteins and lipids are reported to play key roles in the regulation of pathways of the immune system and have been widely studied due to the necessity of the aquafeed industry to replace dietary fishmeal (FM) and fish oil (FO) [14].

Polyunsaturated fatty acids (PUFA) are involved in the regulation of both innate and adaptive immunity, and the inflammatory response through four potential mechanisms including gene expression, eicosanoid metabolism, cellular signalling and membrane organization [15]. Specific transcription factors are activated by a number of PUFA, long-chain (LC)-PUFA and eicosanoid ligands [16], and subsequently regulate the expression of genes related with inflammatory, B-cell and T-cell responses, which play important roles in viral infections. The roles of PUFA in cell membrane organization and signalling pathway mechanisms have been widely studied in humans [15]. Inhibition of T-cell signalling pathways by PUFA is mainly linked to the suppression of the elevation of cytoplasmic calcium concentration through the phosphatidylinositol (PI) signalling system, which is a key event in T-cell activation [17]. Eicosanoids are key mediators of inflammation and the regulation of T and B lymphocyte functions. Arachidonic acid (20:4n-6; ARA) and eicosapentaenoic acid (20:5n-3; EPA), released from membrane phospholipids through the action of phospholipases, are converted to pro- or anti-inflammatory eicosanoids, respectively and, although the enzymes of eicosanoid metabolism have a preference for ARA, increased levels of EPA in the membranes of immune cells inhibit the production of pro-inflammatory eicosanoids [18]. Studies have demonstrated that LC-PUFA have similar roles in immune modulation in fish. Substitution of dietary FO by vegetable oil (VO), with consequent reduction in the ratio of n-3/n-6 LC-PUFA, promotes the synthesis of pro-inflammatory eicosanoids [19, 20], and alters humoral immunity and the expression of pro-inflammatory cytokine genes [21]. Most studies have been performed *in vitro* [22–24], and so little is known about the roles of LC-PUFA during viral infections in fish.

In a previous study, the use of functional feeds led to a milder inflammatory response and reduced severity of heart lesions in salmon experimentally infected by Atlantic salmon reovirus (ASRV), the causal agent of heart and skeletal muscle inflammation (HSMI) [25]. As both HSMI and CMS have similar symptoms, we have employed a similar strategy in the present study to investigate the effects of functional feeds in controlling CMS. The functional feeds contained increased EPA levels, from 3.6% in a reference feed to 14%, and reduced dietary lipid, from 31% to 18% in the functional feeds. Supplementation of histidine was also assessed in one of the functional feeds as this amino acid and related compounds, such as N-α-acetyl-histidine (NAH), have important roles in muscle pH buffering [26, 27] and tissue antioxidant systems [28, 29]. In particular, histidine has been associated with a cardio-protective role in human studies, being potentially beneficial in the alleviation of oxidative stress associated with viral diseases [30]. Thus, we hypothesized that dietary supplementation of this amino acid could have a potentially beneficial effect in fish suffering CMS.

Fish were fed the experimental feeds for 8 weeks prior to infection by PMCV and throughout the study post-infection. The incorporation of LC-PUFA into membrane phospholipids of heart, the main organ affected by the disease, was assessed, and both heart and liver tissues were subjected to histological evaluation. The inflammatory and immune status in heart tissue was assessed after infection by determining gene expression through oligonucleotide microarray analysis.

## Results

### Lipid and fatty acid composition of heart tissue

**Figure 1:**
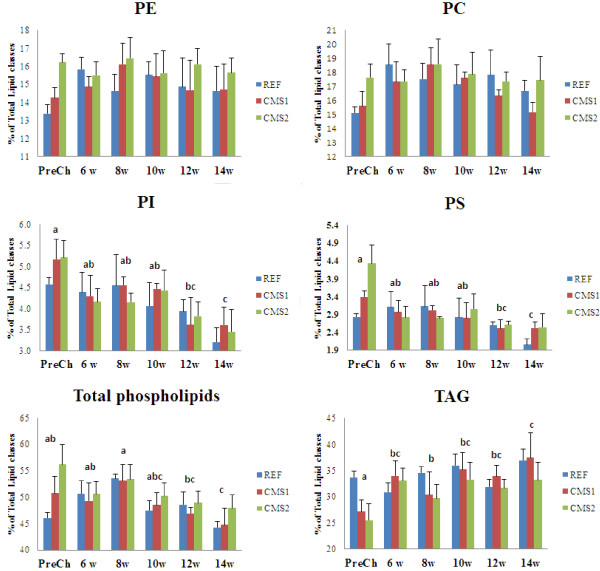
**Proportions of lipid classes in total lipid of heart tissue from fish fed the reference (REF) and functional (CMS1 and CMS2) feeds at different times before (PreCh) and after (6, 8, 10, 12 and 14 weeks) infection with PMCV.** Data were analysed by 2-way ANOVA with “time-course” and “diet” as the two factors. Different letters above time-points indicate significant differences between time-points over the time-course of the infection. The factor diet showed significant differences in PC, PE, total phospholipids and TAG (not indicated). PC, phosphatidycholine; PE, phosphatidylethanolamine; PI, phosphatidylinositol; PS, phosphatidylserine; TAG, triacylglycerol.

The total lipid content of heart tissue was unaffected by dietary treatment and did not show any significant changes over the course of infection (Table 1). Proportions of triacylglycerol (TAG) were lower and those of total PL and the major PL classes, phosphatidylcholine (PC) and phosphatidylethanolamine (PE), significantly higher in heart tissue of fish fed the functional feeds compared with fish fed the REF diet (2-way ANOVA p-value diet, < 0.05) (Figure 1). These differences were particularly apparent at the end of the pre-feeding phase and, after PMCV infection, PL levels generally decreased, significantly for phosphatidylinositol (PI) and phosphatidylserine (PS), over the course of the infection.

**Table 1:** **Fatty acid compositions (percentage of total fatty acids) of total phospholipids of heart tissue from fish fed the reference (REF) and functional (CMS1 and CMS2) feeds at different times before (PreCh) and after (6, 8, 10, 12 and 14 weeks) infection with PMCV**

	PreCh			6 wpc			8 wpc			10 wpc			12 wpc			14 wpc			TWO WAY ANOVA ***P-value***		
	REF	CMS1	CMS2	REF	CMS1	CMS2	REF	CMS1	CMS2	REF	CMS1	CMS2	REF	CMS1	CMS2	REF	CMS1	CMS2	Diet	Week	Diet*Week
**Saturated**	25.9 ± 0.8	31.2 ± 0.9	31.9 ± 0.8	27.6 ± 0.8	29.7 ± 0.7	30.1 ± 0.6	27.2 ± 0.6	29.9 ± 0.7	29.2 ± 0.5	27.1 ± 0.5	29.5 ± 0.3	29.9 ± 0.5	27.6 ± 1.5	30.6 ± 1.4	31.2 ± 1.0	27.9 ± 0.5	31.3 ± 0.4	30.4 ± 0.7	0.000	0.005	ns
**18:1n-9**	13.2 ± 0.6	7.4 ± 0.6	6.8 ± 0.3	9.5 ± 0.4	6.8 ± 0.2	7.2 ± 0.4	10.1 ± 0.3	7.2 ± 0.2	8.0 ± 0.4	10.2 ± 0.8	7.5 ± 0.6	7.5 ± 0.7	11.0 ± 1.5	7.3 ± 0.1	7.6 ± 0.3	11.5 ± 0.6	7.5 ± 0.2	7.3 ± 0.5	0.000	0.003	0.003
**Monounsat**	24.6 ± 2.6	15.4 ± 0.9	15.3 ± 0.7	18.0 ± 1.0	14.7 ± 0.8	15.1 ± 1.1	19.5 ± 0.7	14.9 ± 0.7	16.8 ± 0.9	18.9 ± 1.6	15.6 ± 1.3	15.8 ± 1.9	20.4 ± 3.0	15.5 ± 0.4	15.8 ± 0.7	21.5 ± 1.4	15.7 ± 0.6	15.5 ± 1.0	0.000	0.033	0.065
**18:2n-6**	4.3 ± 0.4	1.9 ± 0.1	1.7 ± 0.1	3.3 ± 0.1	1.7 ± 0.1	1.7 ± 0.1	3.4 ± 0.1	1.8 ± 0.1	2.1 ± 0.2	3.3 ± 0.2	1.8 ± 0.2	1.8 ± 0.1	3.4 ± 0.4	1.7 ± 0.0	1.7 ± 0.1	3.5 ± 0.2	1.8 ± 0.1	1.8 ± 0.1	0.000	0.008	0.021
**20:3n-6**	0.6 ± 0.0	0.1 ± 0.0	0.2 ± 0.0	0.6 ± 0.0	0.2 ± 0.0	0.2 ± 0.0	0.6 ± 0.0	0.3 ± 0.0	0.3 ± 0.0	0.5 ± 0.0	0.3 ± 0.0	0.3 ± 0.0	0.4 ± 0.0	0.3 ± 0.0	0.3 ± 0.0	0.4 ± 0.0	0.2 ± 0.0	0.2 ± 0.0	0.000	0.000	0.000
**20:4n-6**	1.4 ± 0.0	1.7 ± 0.1	1.6 ± 0.1	1.5 ± 0.1	1.7 ± 0.1	1.7 ± 0.1	1.5 ± 0.0	1.7 ± 0.1	1.6 ± 0.0	1.4 ± 0.1	1.7 ± 0.0	1.7 ± 0.2	1.3 ± 0.1	1.8 ± 0.1	1.7 ± 0.1	1.4 ± 0.1	1.8 ± 0.1	1.8 ± 0.1	0.000	0.056	0.002
**n-6 PUFA**	7.3 ± 0.4	4.7 ± 0.2	4.5 ± 0.1	6.2 ± 0.1	4.5 ± 0.1	4.5 ± 0.1	6.2 ± 0.1	4.7 ± 0.0	4.8 ± 0.2	6.1 ± 0.2	4.8 ± 0.2	4.7 ± 0.1	6.0 ± 0.4	4.6 ± 0.1	4.6 ± 0.1	6.4 ± 0.2	5.0 ± 0.1	4.9 ± 0.1	0.000	0.000	0.000
**18:3n-3**	1.5 ± 0.1	0.5 ± 0.1	0.4 ± 0.0	1.1 ± 0.0	0.4 ± 0.0	0.4 ± 0.0	1.2 ± 0.1	0.5 ± 0.0	0.6 ± 0.1	1.3 ± 0.1	0.5 ± 0.0	0.5 ± 0.0	1.3 ± 0.2	0.4 ± 0.0	0.4 ± 0.0	1.4 ± 0.0	0.5 ± 0.0	0.5 ± 0.0	0.000	0.000	0.001
**20:5n-3**	6.3 ± 0.3	9.7 ± 0.3	9.6 ± 0.4	7.7 ± 0.3	10.6 ± 0.3	10.4 ± 0.3	8.0 ± 0.3	10.5 ± 0.2	10.2 ± 0.3	8.4 ± 0.4	11.3 ± 0.4	10.8 ± 0.4	8.0 ± 0.1	10.9 ± 0.6	10.4 ± 0.2	7.7 ± 0.3	10.3 ± 0.4	11.0 ± 0.6	0.000	0.000	0.038
**22:6n-3**	31.5 ± 1.4	33.8 ± 1.3	33.9 ± 1.5	35.8 ± 1.4	35.3 ± 0.9	34.8 ± 1.5	34.6 ± 0.7	34.8 ± 0.5	33.6 ± 0.7	34.5 ± 0.8	33.2 ± 1.9	33.6 ± 1.5	33.4 ± 2.7	32.9 ± 1.2	32.8 ± 1.4	31.7 ± 1.5	32.4 ± 0.5	32.7 ± 1.0	ns	0.000	ns
**n-3 PUFA**	42.0 ± 1.4	48.3 ± 1.7	48.0 ± 1.8	47.4 ± 1.2	50.7 ± 0.8	50.0 ± 1.6	46.8 ± 0.8	50.3 ± 0.8	48.8 ± 0.8	47.3 ± 1.2	49.8 ± 1.7	49.4 ± 1.8	45.7 ± 2.5	48.9 ± 1.8	48.1 ± 1.5	43.8 ± 1.6	47.5 ± 0.5	48.8 ± 1.6	0.000	0.000	ns
**PUFA**	49.3 ± 0.8	53.0 ± 0.9	52.6 ± 0.7	53.7 ± 1.1	55.2 ± 0.8	54.5 ± 1.6	53.0 ± 0.8	55.0 ± 0.8	53.6 ± 0.6	53.4 ± 1.0	54.6 ± 1.5	54.0 ± 1.8	51.7 ± 2.2	53.5 ± 1.7	52.6 ± 1.5	50.2 ± 1.5	52.5 ± 0.6	53.7 ± 1.5	0.000	0.000	ns
**EPA/ARA**	4.6 ± 0.2	5.8 ± 0.4	5.8 ± 0.3	5.2 ± 0.2	6.3 ± 0.2	6.3 ± 0.3	5.5 ± 0.2	6.1 ± 0.0	6.5 ± 0.3	6.0 ± 0.2	6.6 ± 0.1	6.6 ± 0.4	6.1 ± 0.5	6.2 ± 0.3	6.3 ± 0.1	5.7 ± 0.1	5.7 ± 0.4	6.0 ± 0.3	0.000	0.000	0.001
**n-3/n-6**	5.7 ± 0.2	10.3 ± 0.5	10.6 ± 0.3	7.6 ± 0.3	11.2 ± 0.3	11.2 ± 0.5	7.5 ± 0.2	10.6 ± 0.2	10.1 ± 0.5	7.7 ± 0.4	10.4 ± 0.8	10.6 ± 0.3	7.7 ± 1.0	10.6 ± 0.6	10.5 ± 0.3	6.9 ± 0.4	9.6 ± 0.1	10.1 ± 0.5	0.000	0.000	0.066
**Total lipid**	2.8 ± 0.3	3.5 ± 0.2	3.6 ± 0.2	3.32 ± 0.2	3.34 ± 0.2	3.49 ± 0.2	3.8 ± 0.4	3.34 ± 0.2	3.63 ± 0.6	3.84 ± 0.3	3.55 ± 0.2	3.58 ± 0.3	3.85 ± 0.2	3.56 ± 0.1	3.46 ± 0.1	3.81 ± 0.3	3.44 ± 0.5	3.46 ± 0.4	ns	ns	ns

ARA, arachidonic acid; DHA, docosahexaenoic acid (DHA); EPA, eicosapentaenoic acid; PUFA, polyunsaturated fatty acid.

Diet*Week indicates the p value of the interaction of those two factors in the 2-way ANOVA statistical analysis.

The fatty acid compositions of total PL of heart tissue of fish fed the functional feeds reflected the composition of the diets showing different proportions of the LC-PUFA that could potentially influence the immune response [31] (Table 1). Thus, the proportions of EPA, ARA and the EPA/ARA ratio were significantly higher in fish fed the functional feeds compared to fish fed the REF diet (P value diet, <0.05). However, levels of docosahexaenoic acid (22:6n-3; DHA) were similar between the different dietary groups, which was consistent with our previous study using similar feeds in which tissue levels of DHA were more conserved despite differences in the composition of the diets [25]. The proportions of the inflammation-related LC-PUFA in heart total PL changed during the time course of the infection but were similar in fish fed the three dietary treatments (Table 1). The changes in ARA post-infection were not significant, but the proportions of EPA and DHA were significantly higher at 6-wpc compared with pre-challenge. After 6-wpc, levels of DHA progressively decreased during the time-course of the infection but proportions of EPA were significantly higher at 10-wpc. As a consequence of the latter, the EPA/ARA ratio in heart total PL was also higher in all dietary treatments at 10-wpc. There were no differences in the EPA/ARA ratio in heart PL between fish fed the different diets in the later stages of the infection.

### Heart and liver tissue histopathology

**Figure 2:**
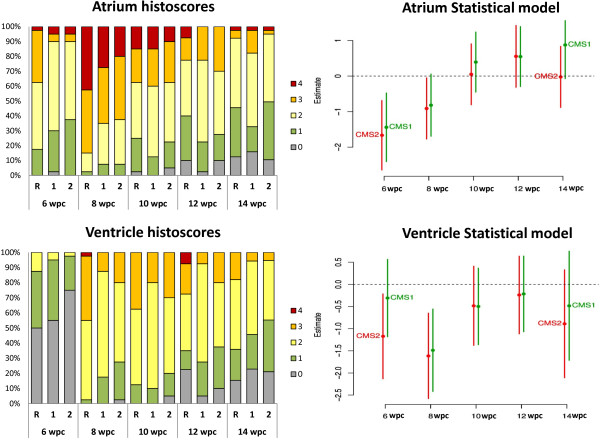
**Histopathology results in heart tissue.** Figures on the left are representing incidence (percentage of fish sampled) and severity of histopathology (based on the criteria described in methods) in both parts of the heart, atrium and ventriculum at 6-, 8-, 10-, 12-, 14-weeks post-challenge in fish fed the Reference diet (R), and the two functional feeds CMS1 (1) and CMS2 (2). Figures on the right are representing the statistical analysis of the atrium and ventriculum histoscores. Estimated effects of CMS1 and CMS2 diets in comparison to the REF diet by sampling weeks. Negative estimates mean there are lower scores and positive that there are higher scores than for the REF dietary group. Error bars denote approximate 95% confidence limits.

Histological changes were consistent with CMS and first observed in the atrium at 6-wpc (Figure 2), characterized by focal infiltration of inflammatory cells dominated by lymphocytes. A degenerative process associated with the inflammatory changes was also observed in cardiomyocytes of the atrium. The most marked increase in histopathological scores was found in the ventricle of fish from all the dietary groups from 6- to 8-wpc. These changes were typified by multifocal infiltration of inflammatory cells, dominated by lymphocytes and macrophages, and concomitant degeneration and necrosis of myocytes. Another typical finding was hyperplasia of endothelial cells in inflamed areas. Inflammatory changes were greatest in the atrium from 6- to 8-wpc and significantly higher for all groups at both time points. By 10-wpc, the inflammatory changes and myocyte necrosis had levelled off in all groups, and was not different from 8-wpc. At 12- and 14-wpc there was a moderate decline in inflammatory scores (Figure 2).

Differences between dietary groups were found mainly in the early stages of the disease, at 6- and 8-wpc. Fish fed both functional feeds showed significantly lower histoscores in the atrium at both 6- and 8-wpc compared to fish fed the REF diet (Figure 2). Lesions in the ventricle were also lower in fish fed the functional feeds, being significant in both dietary groups at 8-wpc but only for the fish fed with the CMS2 diet at 6-wpc. There were no differences in inflammation in either part of the heart between dietary groups at 10- and 12-wpc. At the end of the trial, 14-wpc, lesions in the atrium were not significantly different between groups, although fish fed with CMS2 had lower histoscores compared with the other groups. In ventricle, fish fed both functional feeds showed lower histoscores although they were not significantly different (Figure 2).

**Figure 3:**
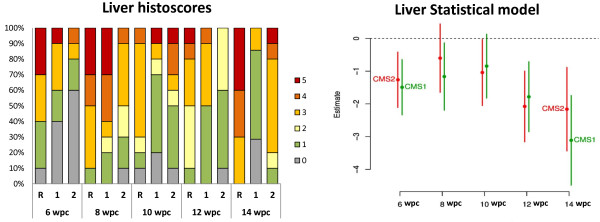
**Histopathology results in liver tissue.** Figure on the left is representing incidence (percentage of fish sampled) and severity of histopathology (based on the criteria described in methods) at 6-, 8-, 10-, 12-, 14-weeks post-challenge in fish fed the Reference diet (R), and the two functional feeds CMS1 (1) and CMS2 (2). Figure on the right is representing the statistical analysis of liver histoscores. Estimated effects of CMS1 and CMS2 diets in comparison to the REF diet by sampling weeks. Negative estimates mean there are lower scores and positive that there are higher scores than for the REF dietary group. Error bars denote approximate 95% confidence limits.

The liver histology scores, based on the degree of steatosis were interpreted as vacuole-formation in hepatocyte cytoplasm characterized by both micro- and macro-vesicular lesions. There were clear differences between the fish fed the functional feeds and fish fed the REF diet at all samplings points post-challenge, with the latter group presenting higher micro- and macro-vesicular steatosis. Severity of the liver histopathology was significantly higher at the beginning of the infection and greater at the end of the challenge (12- and 14-wpc) in salmon fed the REF diet compared to fish fed the two functional feeds (Figure 3).

### Viral load

**Figure 4:**
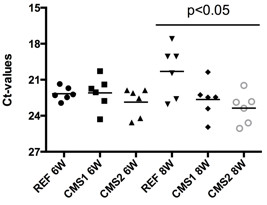
**Viral load in heart tissue of the different dietary groups at 6- and 8-weeks post-challenge.** Viral load was determined by quantitative real-time PCR analysis of Piscine Myocarditis Virus. Results are presented as CT values (normalized) as a basis for showing the relative level of virus expression (n = 6) Levels of significant differences (*t*-test) were calculated using the relative expression software tool (REST 2009).

Viral load was similar for the three dietary groups at 6-wpc, however by 8-wpc, although individual variation in viral RNA was observed, heart viral load was higher in fish fed the REF diet compared with fish fed the functional feeds, significantly so in the case of fish fed CMS2 (Figure 4).

### Cardiac transcriptomic responses

#### Time-course of disease

**Figure 5:**
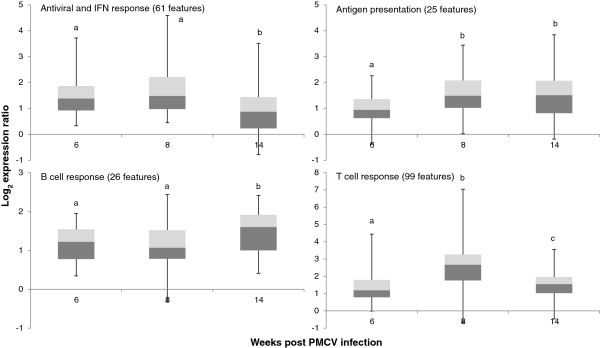
**Time-course of four immune pathways to PMCV infection examined with microarray in salmon fed with REF diet.** Boxes show median log_2_ gene expression ratio (total number of features for each pathway indicated in panel header) between pre-challenge and time points post infection, with the 25^th^ (dark grey) and 75^th^ (light gray) percentile, and whiskers indicate minimum and maximum values. Letters indicate significant differences between time points (p < 0.05, Student *t*-test).

Comparison of gene expression changes in fish fed the REF diet over the course of infection (6-, 8- and 14-wpc) relative to pre-challenge levels showed highly co-ordinated up-regulation of genes involved in immune pathways (Figure 5); data for representative genes included in Figure 5 are in Table 2. The antiviral and IFN response (62 features), mainly represented by previously annotated virus-responsive genes [32], showed highest expression at 6- and 8-wpc that levelled off by 14-wpc. The largest group of genes associated with T cell responses (99 features) reached peak levels by 8-wpc coinciding with the peak of cardiac histoscores. A similar profile was observed for genes involved in antigen presentation via major histocompatibility complexes (MHC class I and II), however their induced expression remained stable to 14-wpc. The B cell response, represented by various immunoglobulins (26 features), showed increasing expression from 6- and 8-wpc until peak levels at 14-wpc. Recovery in cardiac histopathology by 14-wpc was consistent with significantly reduced expression of several inflammatory markers, such as *granzyme A*, *serum amyloid A*, *TNF decoy receptor* (*tnfrsf6b*) and *neutrophil cytosolic factor 4*. It is also noteworthy that negative immune regulators such as *suppressor of cytokine signaling 3* and *lymphocyte antigen 75/cd205* were induced by 14-wpc.

**Table 2:** **Examples of immune genes induced in CMS challenged salmon. Data are challenge to pre-challenged expression ratios (fold changes) on the fish fed with the REF diet**

Genbank	Gene, symbol	6 wpc	8 wpc	14 wpc
	*Antigen presentation and VRG*			
**U20945**	MHCII beta chain	4.80	10.91	8.10
**DY713447**	CD40	1.73	3.06	3.26
**AF508864**	MHC class I antigen	3.60	6.17	10.80
**DW538400**	IFN-induced protein 44, ifit44	6.93	24.09	6.77
**117530907**	Barrier-to-autointegration factor, bainf	13.20	14.39	2.96
**EG795847**	GTPase IMAP family member 7, gimap7	3.65	9.05	3.44
	*T cells*			
**AJ841811**	Interferon gamma, ifng	3.60	8.03	1.29
**DY710412**	Granzyme A, graa	21.78	131.75	6.62
**NM_0011235**	CD8 alpha	3.13	12.06	6.44
**213070237**	CD45	7.11	10.20	11.83
**EG819885**	Cytotoxic T-lymphocyte protein 4, ctla	5.18	14.95	3.04
	*Inflammatory markers, regulators*			
**CX032230**	TRAF interacting protein, traip	1.26	2.32	3.27
**117427898**	Chemokine CCL-C24	2.98	10.93	16.14
**AJ517803**	TNFR associated factor 3, traf3	2.70	1.29	3.15
**CU652893**	Serum amyloid A5, saa	3.11	1.76	1.53
**EG881931**	TNF decoy receptor, tnfrsf6b	3.54	10.64	1.41
**EG904168**	IL1b	2.48	2.66	2.84
**117496064**	Neutrophil cytosolic factor 4, ncl4	1.43	2.64	0.94
**213077403**	TNF alpha-induced protein 2, tnfaip2	3.39	5.28	3.95
**S48406409**	Lymphocyte antigen 75, Ly75/CD205	2.21	2.64	9.20
**213081931**	Helicase lymphoid-specific, hells	1.03	6.06	6.52
**DW578491**	Suppressor of cytokine signaling 3b, socs 3b	1.34	1.42	3.41

### Effects of functional feeds pre- and post-challenge

#### Effects on immune responses

**Figure 6:**
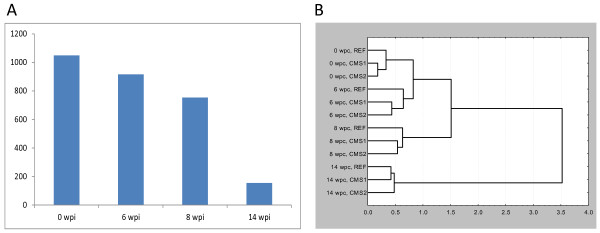
**Effect of functional feeds on gene expression in heart tissue of Atlantic salmon examined with microarray. A**: Number of differential expressed genes (y-axis) per time point post infection (x-axis). **B**: Hierarchical clustering by 2419 features (Pearson r, Ward’s method)

**Figure 7:**
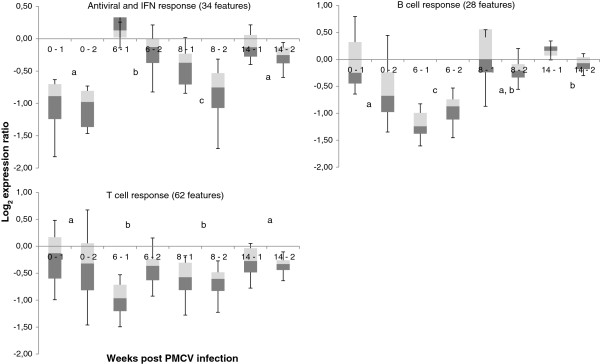
**Effect of the two functional feeds on immune gene expression in heart tissue of Atlantic salmon pre (week 0) and post (weeks 6, 8, 14) PMCV infection, as examined with microarray.** Data are log_2_ expression ratios between functional feeds (CMS1 and 2, noted respectively as -1 and -2 after each time points) and reference feed (REF). Boxes show median values (total number of features for each pathway indicated in panel header) with the 25^th^ (dark grey) and 75^th^ (light gray) percentile, and whiskers indicating minimum and maximum values. Letters indicate significant differences between time points (p < 0.05, Student *t*-test).

A large fraction of differentially expressed genes encoded proteins of the immune system and genes from several functional groups showed similar expression profiles over the course of the infection (Figure 6; Additional file 1: Table S1); results for selected genes are given in Table 3. Overall, the functional feeds resulted in down-regulated levels of immune genes, suggestive of immune suppressive actions (Figure 7). At pre-challenge this was most significant for antiviral and IFN responses, which showed reduced expression in fish fed CMS1 and CMS2 compared to fish fed the REF diet (Figure 7). This down-regulation was also significant at 8-wpc, although with lower magnitude, and this coincided with the most significant reduction in ventricle histoscore in fish fed the functional feeds (Figure 2). Among antiviral and IFN genes, the largest expression differences were found in genes known for potent responses to viruses, such as *very large inducible GTPase 1*, *IFN inducible MX protein* and *receptor-transporting protein 3*. Notable exceptions were two genes encoding IFN regulatory factors – IRF4 and IRF7 – that had from 2.6 to 4.4-fold higher expression before challenge in salmon fed with the functional feeds compared to the REF diet. The expression profiles of immunoglobulin (Ig) genes most likely reflected the influx and amount of B cells in the heart. Fish fed the functional feeds had slightly lower abundance of 28 Ig transcripts before challenge that were further reduced by 6-wpc, followed by normalized levels from 8- to 14-wpc. The most striking immunosuppressive effect of functional feeds was observed for T cell response genes, as suggested by significantly reduced expression of 64 transcripts at pre-challenge, 6- and 8-wpc, and moderately lower reduction at 14-wpc. Down-regulation of *CD8 beta*, *granzyme A/K* and *IFN gamma* could imply that a large fraction of this population was represented by cytotoxic T cells. *Granzyme A* and *TNF decoy receptor tnfrsf6b*, which were also down-regulated by the functional feeds, showed strongest correlation with heart histopathology level in a previous CMS study [11].

**Table 3:** **Examples of immune genes that responded to the functional feeds**

Genbank Or Probename	Gene, Symbol	0 wpc		6 wpc		8 wpc		14 wpc	
		CMS1	CMS2	CMS1	CMS2	CMS1	CMS2	CMS1	CMS2
	***Antiviral responses***								
**BQ035726**	Very large inducible GTPase 1-3, vlig	−3.71	−1.87	−1.30	−2.75	−3.53	−4.80	−1.51	−3.71
**?**	IFN inducible mx protein	−5.37	−2.61	1.23	0.78	−1.77	−4.04	−1.09	−1.08
**DY741158**	STAT2	−2.70	−4.19	1.26	1.03	1.06	−1.67	1.20	1.08
**CB500614**	Pyrin, bty	−1.38	−2.76	1.12	−1.03	−2.39	−3.50	−1.79	−1.14
**BX909789**	IRF4	2.82	2.64	3.28	1.46	−1.73	−2.14	1.31	−1.06
**213061919**	IRF7	3.33	4.43	−1.01	−1.19	1.54	−1.18	1.27	1.45
**DW538275**	Receptor-transporting protein 3	−1.90	−2.31	−0.09	−0.43	−0.85	−1.17	0.45	−0.13
	***T cells***								
**Ssa#TC108524**	MHC class I antigen	−2.33	−1.57	−0.36	−1.02	−0.05	−0.34	−0.33	−0.52
**?**	IFNg	−1.66	−1.09	−2.18	−1.98	−2.44	−1.99	−1.32	−1.91
**AY693394**	CD8 beta	1.19	−1.35	−2.07	−1.76	−2.02	−1.43	−1.60	−1.74
**CA368982**	Kidney injury molecule 1, havcr1	1.36	−1.31	−1.46	−1.64	−4.27	−2.62	−1.36	1.19
**DY710412**	Granzyme A-1, graa	−2.24	1.30	−2.65	−2.18	−3.04	−2.94	−1.04	−2.07
**?**	Granzyme A-2, graa	−1.48	−1.11	−2.76	−3.27	−3.56	−2.50	−1.19	−2.61
**CR369847**	NF activated T-cells calcineurin-dependent 1, nfatc1	−1.10	2.27	−1.63	−1.03	4.62	3.09	−1.88	−1.35
	***Inflammatory markers, regulators***								
**213077403**	TNF alpha-induced protein 2, tnfaip2	2.53	2.48	−3.51	−1.35	−2.53	−2.29	2.03	1.11
**EG871595**	c3a anaphylatoxin chemotactic receptor, c3ar1	−1.02	1.53	1.12	1.13	−2.53	−1.84	−1.17	−1.77
**DW576911**	Cytochrome P450 family 2 subfamily V, cyp2u1	−1.90	1.13	−5.38	−2.73	−1.74	−2.67	−2.35	−1.54
**213081824**	FcRgamma-like protein, fcer1g	−6.40	−3.06	−2.44	1.16	−3.18	−2.05	1.20	−2.59
**EG881931**	TNF decoy receptor, tnfrsf6b	1.36	1.13	−1.73	−1.69	−2.96	−3.18	1.21	−1.10
**213081931**	Helicase lymphoid-specific, hells	2.24	4.47	1.34	2.20	−2.25	−1.98	−1.06	1.41
**CK888744**	fetuin B	1.16	1.39	0.87	1.19	1.42	0.58	1.51	−0.56
**S48406409**	Ly75/CD205	2.79	2.75	−1.13	−1.31	2.06	1.55	−1.27	−1.34

Data are expression ratios (fold changes) of fish fed the functional feeds (CMS1 and CMS2) relative to the REF diet during the course of the infection.

### Effects on lipid metabolism

**Figure 8:**
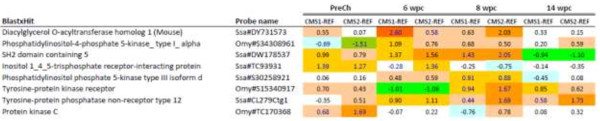
**Expression between functional feeds (CMS1 and CMS2) and reference diet (REF) of genes related with phosphatidyl inositol signalling pathway.** At cut off log2-ER = 0.8 (1.75-fold). Red/orange colour intensity indicates higher expression and green/blue colour intensity indicates lower expression.

**Figure 9:**
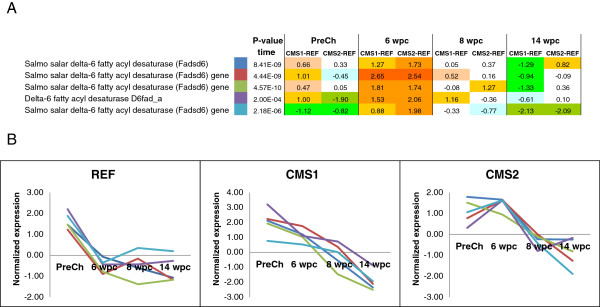
**Expression of fatty acyl desaturase (Fadsd6) gene. A)** Normalized expression of different probes of the oligoarray from delta-6 fatty acyl desaturase (Fadsd6) gene over the time course of the PMCV infection on fish fed with the REF diet and the functional feeds (CMS1 and CMS2). **B)** Expression ratios (fold changes) of different probes of the oligoarray from delta-6 fatty acyl desaturase (Fadsd6) gene. Data are fish fed the functional feeds (CMS1 and CMS2) relative to the REF diet during the course of the infection. Red/orange colour intensity indicates higher expression and green/blue colour intensity indicates lower expression.

Differences in expression of genes involved in lipid metabolism that could potentially modulate the immune response were also observed over the course of the infection (Additional file 1: Table S1). Among the significantly relevant genes were those related to the PI signalling system (Figure 8) and the biosynthesis of LC-PUFA. In general, the expression of the genes related to the PI signalling pathway was not correlated with tissue pathology or the expression of immune-related genes, as there was higher activation of those genes at all sampling points after PMCV infection in fish fed the functional feeds compared with fish fed the REF diet. Of the lipid metabolism genes (Additional file 1: Table S1), the most significant effect was detected with probes for the *delta-6 fatty acyl desaturase (Fadsd6)* gene (Figure 9A). Differences between the three dietary groups were most prominent at 6-wpc, when expression of this gene was higher in the fish fed with the functional feeds compared with fish fed the REF diet. However, these differences appeared to correlate more with heart tissue pathology rather than diet as the expression of this gene significantly decreased over the time course of the infection in all dietary groups (Figure 9B).

## Discussion

The present study demonstrated the potential of dietary immunomodulation for reducing the pathological outcome of virus-associated heart diseases in salmon. Specifically, increased dietary levels of EPA and reduced lipid content were associated with altered expression of genes related with the immune response after an infection with PMCV, significantly reduced pathology in heart and liver tissue, and reduced viral loads at 8 wpc when there was a peak in heart pathology. In contrast, the addition of histidine did not appear to improve the performance of the fish as the level of heart lesions and the expression of genes related with the immune response were not further reduced in fish fed the histidine supplemented diet.

The specific mechanism explaining the potential role(s) of reduced dietary lipid content in the effects of the functional feeds is not clearly established [25, 33]. Previous studies on HSMI showed that there was reduced lipid deposition (steatosis) in livers of fish fed the functional feeds with lower lipid content at initial stages of the disease and the steatosis was usually more frequent when the severity of the heart lesions was high [33]. Thus, there was some association between liver lipid metabolism and on-going viral infection, although earlier studies had found no correlation between the severity of heart and liver lesions during different stages of HSMI disease, associating the liver lesions to the circulatory disturbances as a consequence of heart pathology [34]. Therefore, although the precise mechanism of the reduced liver steatosis index at initial stages in fish fed the functional feeds was not identified in detail, lower dietary lipid was a likely contributing factor [33]. The functional feeds used in the present study were beneficial for the prevention of liver pathology associated with CMS. Fish fed functional feeds showed lower liver histoscores over the whole course of the infection, significantly so at 6-, 12- and 14-wpc, possibly due to the lower dietary lipid content that could reduce liver lipid metabolism when circulatory disturbances are potentially affecting this organ.

Levels of EPA and ARA, and the EPA/ARA ratio, in heart tissue PL were significantly altered by the functional feeds, and hence one of the main strategies of the experimental design was achieved. Specifically, the potential bioavailability of EPA and its proportion relative to ARA was always higher over the time-course of the infection in fish fed the functional feeds compared with fish fed the REF diet. Interestingly, the use of Southern hemisphere FO in the functional feeds in the present trial also increased the level of ARA in heart tissue PL and thus the EPA/ARA ratio was actually lower than in heart tissue of fish in the previous trial on HSMI [25]. The relative proportions of ARA and EPA remained generally constant during the time-course of the PMCV infection although, as described above for DHA, there were increased levels of both ARA and EPA when, according to gene expression analysis, there was activation of the immune response. However, this was only significant for EPA, possibly reflecting the higher absolute levels of EPA generated by the functional feeds in the present trial.

As previously described in humans [35] and fish [21, 36, 37], the fatty acid compositions of membrane PL play a critical role in the regulation of the innate and adaptive immune response. Fatty acids such as ARA, EPA and DHA, released from membrane PL through the action of phospholipases, modulate the immune response not only through eicosanoids, but also by modifying signalling pathways and stimulating immune-related nuclear transcription factors (PPARs and NFkβ) and the production of cytokines [15]. The n-6 LC-PUFA, ARA, is associated with pro-inflammatory responses as the precursor of pro-inflammatory eicosanoids, and EPA, as well as being the precursor of anti-inflammatory eicosanoids, can also decrease the production of ARA-derived eicosanoids through competition for the eicosanoid-synthesising enzymes. DHA has also an anti-inflammatory role in humans, particularly relevant at the recovery phase of an inflammatory process, as it is a precursor of immune-resolving resolvins and protectins [31].

Although macrophages, primarily produced in the head kidney of fish, are a major source of eicosanoids, production of eicosanoids at the site of the infection (e.g. heart in CMS) is also highly relevant due to the short life of these LC-PUFA derivatives [38]. As previously reported in unchallenged [36] and challenged fish [25], DHA levels in heart tissue PL did not reflect dietary levels, being generally similar in fish fed the three diets in the present study. This may reflect the fundamental role of DHA in the maintenance of cellular membrane structure and fluidity [39]. In our previous study investigating the effects of similar diets on HSMI, levels of DHA were not significantly affected during the time-course of the infection after challenge with ASRV [25]. In contrast, the levels of DHA in heart tissue PL changed significantly over the time-course of the infection after PMCV challenge. Levels of DHA were higher when there was enhanced immune response and the heart lesions were more prominent. Whether DHA levels were related with the activation of immune pathways involved in the control of the inflammatory process requires further investigation in fish. However, the present results suggest that changes in the levels of DHA may be associated with PMCV infection and therefore this fatty acid could have a role in the immune response in fish as has been described in humans [31]. The associations between levels of ARA, EPA and DHA in heart PL, the extent of heart histological lesions, and cardiac expression of genes related to the immune response, indicated that dietary effects on incorporation of LC-PUFA into cell membranes of affected tissues could have important immunomodulatory roles in viral infections.

The relevance of dietary supplementation with EPA was supported when evaluating the expression of a key enzyme of LC-PUFA biosynthesis, *delta-6 fatty acyl desaturase* over the course of the infection*.* Higher expression of this enzyme and potentially higher biosynthesis of n-3 LC-PUFA was reported previously in liver of fish fed similar functional feeds compared with fish fed a reference diet [25], later linked to dietary lipid/energy levels [40]. However, the present study is the first to report a generally decreased expression of this enzyme in all dietary groups over the course of PMCV infection after 6-wpc, possibly indicating a negative effect of the viral infection on LC-PUFA biosynthesis. This highlights the importance of dietary supplementation of anti-inflammatory n-3 LC-PUFA when fish are suffering viral heart disease although further studies are required.

In addition to fatty acid composition, the PL class composition was also affected during viral infection, with decreased proportions of PI and PS as the infection progressed. Phosphorylated derivatives of PI such as phosphatidylinositol 4,5-bisphosphate (PIP_2_) are involved in the production of the intracellular second messengers diacylglycerol (DAG) and inositol 1,4,5-triphosphate (IP_3_) through the action of the phospholipase C [17]. DAG and IP_3_ activate calcium channels, increasing Ca^+2^ concentration in the cytosol, with this being an essential step for the survival of some human viruses [41]. PS has also been related with this signalling pathway as it is an important activator of protein kinase C [42, 43]. The difference in expression of genes related with PI signalling between the dietary groups along with decreased proportions of PI and PS, suggested relevance of this pathway during the infection, and could reflect utilization of these PL classes associated with an immunomodulatory role. This is a novel finding and the composition/content of specific PL classes could be an interesting area for future studies on clinical nutrition in fish.

Diet alone had marked effects on the cardiac expression of immune genes prior to challenge with functional feeds suppressing genes related with innate antiviral responses and even those specific for lymphocytes. Broadly similar effects were recently reported in a trial investigating substitution of dietary FO with VO in salmon [44]. In that study, expression levels of immune-related genes in liver of fish fed a FO diet, with a fatty acid composition similar to the functional feeds, were generally lower than in fish fed a VO diet, with a composition similar to the present REF diet. However, in the earlier study, genes related with the T-cell response were expressed at a lower level in fish fed the VO diet [44]. This may be explained by the higher levels of EPA present in the functional feeds used in the present study compared with the FO diet used in the earlier study, as the influence of EPA in controlling T-cell signalling pathways has been documented previously [15, 18]. Expression of genes related with non-specific immune responses were also higher in grouper (*Epinephelus malabaricus*) fed a VO/FO blend compared with fish fed FO alone [37]. So it appears that, even without any apparent infection, inclusion of FO could dampen both innate and adaptive immune responses in fish. Thus, the generally higher immune status of the fish fed the REF diet could be a factor in the earlier development of inflammation and heart lesions associated with CMS in these fish compared with the fish fed the functional feeds. Put another way, the functional feeds generally lowered the immune/inflammatory status prior to viral challenge and, in doing so, delayed and moderated the response to the infection. This was supported by the recent finding that showed low-responder fish to CMS, which developed high infection levels in the absence of cardiac pathology, had ablated expression of adaptive immunity genes and genes involved in T cell responses [11]. However, it must be emphasised that immune and inflammatory responses are essential components of the host defence against infections [45]. Therefore, strategies modulating the inflammatory response must be a balance between promoting resolution and mitigating negative effects of chronic inflammation that can lead to damaging fibrosis without preventing the initial onset of the response [31].

Interestingly, IFN regulatory factors 4 and 7 were among the few innate antiviral genes that were induced by the functional feeds prior to infection. While IRF7 presumably is an important regulator of the antiviral IFN response in fish, as in mammals [46], the suggested role of IRF4 as a negative regulator of TLR signalling and pro-inflammatory cytokines in mice peritoneal macrophages [47] is interesting in view of the down-regulated antiviral state pre-challenge of fish fed the functional feeds and hence the possible implications for the positive effects post-infection. Whether IRF4 has a similar function in fish has yet to be confirmed.

The time-courses for the development and resolution of histopathology and immune gene responses were similar to those observed previously [10, 11]. In addition, there were correlations between the expression of genes related with immune responses, viral load and heart lesions, in agreement with Timmerhaus et al. [10]. Importantly, however, the temporal differences observed between the different dietary treatments in the present study strengthened these correlations. Thus, the changes in the progression of CMS heart pathology, gene expression and viral load, in fish fed the REF diet were temporally similar to the development of the infection described previously [10], whereas there was a clear delay in the appearance of those changes in fish fed the functional feeds. Although viral load was not monitored at later stages of the PMCV infection, the aforementioned correlation between histopathology and gene expression in the REF group allow us to hypothesize that viral load in fish fed the functional feeds would be higher at the later stages. Therefore, the lower viral load in heart of fish fed the functional feeds at early stages of PMCV infection was probably a direct effect of diet on initial viral replication as reported previously after ASRV infection [25]. Furthermore, in addition to a delay in the appearance of heart pathology in fish fed the functional feeds, a slightly improved performance of fish fed the CMS2 diet was observed, which could be associated with reduced expression of genes associated with the innate immune response.

At 6-wpc, gene expression and heart tissue histopathology of fish fed the REF diet were in agreement with those reported previously [10]. Thus, atrium was affected by the infection at 6-wpc with higher histoscores in fish fed the REF diet compared to fish fed the functional feeds, whereas ventricle was only slightly affected. A peak in the expression of genes related with the complement response, B and T-cell responses, and apoptosis was expected at this time post-infection [10], and many of the genes related to these pathways showed lower expression in fish fed the functional feeds compared to fish fed the REF diet. In addition, genes related with antiviral and interferon responses often showed higher expression in fish fed the functional feeds compared with fish fed the REF diet. The peak in expression of the latter genes was reported to be at 2-4 wpc in the earlier study [10], supporting the conclusion that there was a delayed response in fish fed the functional feeds. A similar delayed response to viral infection of around 2 weeks was observed previously after ASRV infection in salmon fed functional feeds [25].

As the CMS disease progressed the extent of lesions in the heart increased in both atrium and ventricle with the differences between the fish fed the functional feeds and fish fed the REF diet being most pronounced at 8-wpc. At this time-point, fish fed the functional feeds presented lower histoscores in both atrium and ventricle compared with fish fed the REF diet, which could be due to the up-regulation of genes related with host immune response observed at 6-wpc in fish fed the REF diet. At 8-wpc, following the hypothesis of delayed development of CMS in fish fed the functional feeds, higher expression of genes involved in antiviral and innate immune pathways could have been expected in these fish compared with fish fed the REF diet but, in contrast, these groups showed lower activation of these pathways. Dietary modulation of the inflammatory response has, of course, been described previously in fish. Higher incorporation of n-3 LC-PUFA in biological membranes of immune cells led to a lower expression of pro-inflammatory cytokines in gilthead seabream (*Sparus aurata*) [21], decreased antibody production and macrophage killing activity in rainbow trout [48], and lower production of pro-inflammatory eicosanoids in Atlantic salmon [19, 20]. Furthermore, dietary EPA suppressed production and release of TNF, interleukins and IFN in humans [49]. Dietary EPA also suppressed key components of the antiviral response in gilthead sea bream, and dietary VO inclusion modulated the expression of Mx proteins, interferon-induced mediators of innate resistance to RNA virus [50]. According to these previous studies, the increased proportions of dietary EPA in the functional feeds used in the present study could be a factor in preventing an uncontrolled immune response that could be more harmful to the fish.

As expected, many genes related with the T-cell response showed lower expression in fish fed the functional feeds compared with fish fed the REF diet [10]. Although gene expression was not assessed at 10- and 12-wpc, increased transcription of genes involved in virus clearance may be predicted based on histopathology of heart lesions at these time-points. By 14-wpc, there was a clear remission in the lesions in both parts of the heart in all dietary groups and therefore no significant differences in the pathology were observed between them. Even though the list of genes differentially expressed at 14-wpc in fish fed the different diets was shorter than the ones from previous sampling points, some pathways represented in the list of genes differentially expressed at 14-wpc in fish fed the different diets could still indicate a more effective remediation of the inflammatory response by the group of fish fed with the functional feeds. Thus, important markers of the T-cell response such as *T cell receptor alpha, CD8 beta and alpha* and *CD82 antigen* showed lower expression in fish fed the functional feeds, but expression of genes related with PI signalling was higher in those fish compared with fish fed the REF diet. As mentioned above, the PI signalling pathway is intimately involved in the regulation of the T-cell response [17], and dependent on the levels of membrane PL classes and their fatty acid compositions. As the lesions in heart tissue in CMS were associated with the action of CD8+ cytotoxic T-cells [10], factors controlling this response, such as anti-inflammatory n-3 LC-PUFA, could be key to the general better performance observed in the fish fed the functional feeds. Furthermore, results from the previous study evaluating the use of functional feeds in salmon infected with HSMI were consistent with this hypothesis. Thus, milder expression of genes related with inflammatory response and virus clearance, including those related with T-cell response, were reported in salmon fed the functional feeds leading to better performance of those fish over the course of the HSMI infection [25].

## Conclusions

The present study is the first to describe the effects of functional feeds on the expression of genes related with the immune response after infection of Atlantic salmon with PMCV. Significant differences in immune and inflammatory responses and pathology in heart tissue were found in fish fed the different dietary treatments over the course of the infection, highlighting the potential immune modulatory role of dietary fatty acid composition and lipid content in viral infections in salmon. The strategy was effective in significantly reducing heart pathology and also decreasing the time-course of the viral infection. Furthermore, liver pathology was significantly reduced during the time course of the infection in fish fed the functional feeds. Molecular signatures showed lower expression of immune-inflammatory genes, especially those reported to be correlated with heart lesions in the clinical phase (i.e. T cells). Moreover, transcriptome analysis revealed a novel pathway of the fish immune response, the PI signalling pathway, that functional feeds could modulate, as well as potential suppression of LC-PUFA biosynthesis as the infection progresses, stressing the importance of dietary supplementation with n-3 LC-PUFA.

## Methods

### Experimental feeds and fish

Three fishmeal-based diets, based on a previous study investigating HSMI [25], were formulated and manufactured by EWOS Innovation (Dirdal, Norway) (Table 4). The reference diet (REF) was essentially a standard, commercial formulation with 31% lipid with the added oil being a blend of Northern hemisphere FO and rapeseed oil. The two functional feeds (CMS1 and CMS2) both contained a lower level of lipid (18%) that was balanced by increased protein, provided by fishmeal and krill meal. Therefore, as a major factor differing between the REF and functional feeds was dietary lipid level, the feeds were not isolipidic or isoproteic. The added oil in the functional feeds was provided by a Southern hemisphere FO. As a result the CMS1 and CMS2 feeds had a similar fatty acid profile containing higher proportions of EPA (almost 14%) and a n-3/n-6 PUFA ratio around 4, in comparison to the REF feed (<4% EPA and an n-3/n-6 ratio of 1.4) (Table 5). The only major difference between the functional feeds was that CMS1 was supplemented with additional histidine for reasons described above [30].

**Table 4:** **Formulation (g/Kg) and proximate composition (percentage) of the reference (REF) and functional (CMS1 and CMS2) feeds**

Component (g/100 g) ^1^	REF	CMS1	CMS2
Fish meal and hydrolysates	42.1	53.0	53.0
Plant protein concentrates^2^	21.5	18.0	18.0
Northern fish oil	13.0	0.0	0.0
Southern fish oil	0.0	10.0	10.0
Rapeseed oil	10.7	0.0	0.0
Carbohydrate-based binders^3^	11.9	11.9	12.1
Micro premixes^4^	0.8	1.7	1.9
Krill meal^5^	0.0	5.0	5.0
Histidine (synthetic)	0.0	0.4	0.0
Total	100	100	100
**Proximate composition**			
Moisture	6.5	6.5	6.5
Fat	31.0	18.0	18.0
Protein	42.2	53.4	53.4

^1)^ All ingredients sourced from EWOS stocks unless otherwise stated.

^2)^ Includes soy protein concentrate, pea protein concentrate, wheat gluten and sunflower meal.

^3)^ Includes wheat grain.

^4)^ Includes vitamins, minerals, crystalline amino acids, ammonium phosphate.

^5)^ Aker Biomarine AS.

**Table 5:** **Fatty acid compositions (percentage of total fatty acids) of the reference (REF) and functional (CMS1 and CMS2) feeds**

***Fatty acid***	REF	CMS1	CMS2
**Saturated**	19.96	26.62	25.83
**Mounsaturated**	56.64	36.42	36.08
**18:2n-6**	8.38	4.94	5.55
**20:3n-6**	0.15	0.30	0.30
**20:4n-6**	0.18	0.64	0.66
**n-6 PUFA**	9.50	6.91	7.16
**18:3n-3**	3.11	1.27	1.31
**20:5n-3**	3.51	13.66	13.38
**22:6n-3**	4.77	8.41	8.45
**n-3 PUFA**	13.28	27.55	27.79
**PUFA**	23.40	36.97	38.09
**n-3/n-6**	1.40	3.98	3.88
**EPA/ARA**	19.35	21.40	20.20
**% Lipid**	25.60	17.30	16.46

ARA, Arachidonic acid; EPA, eicosapentaenoic acid; PUFA, polyunsaturated fatty acids.

A total of 675 Atlantic salmon (*Salmo salar* L.), SalmoBreed IPN strain (average weight ca.150 g), were distributed into nine tanks (1 m^3^) at the VESO facility, Vikan, Norway and fed one of the three feeds (3 tanks of 75 fish each per dietary treatment) for a period of 8 weeks prior to being transferred to the challenge tanks. Feeding ration was a maximum of 2%. Seawater/brackish water (ca. 25 ‰-35 ‰) delivery was flow-through sufficient to maintain oxygen-satiation in effluent water 70%. Water temperature was maintained at 12 ± 1°C, and a photoperiod 24:0 h light/dark regime was followed. Stocking density of the tanks was a maximum of 60 Kg/m^3^. Feeding and infection challenge trials were approved by The National Animal Research Authority (http://www.fdu.no) according to the ‘European Convention for the Protection of Vertebrate Animals used for Experimental and other Scientific Purposes’ (EST 123).

### Growth performance

Growth performance of fish was assessed using repeated measurements of tank mean weights determined at the start of the trial (day 0), challenge-day (day 92) and then at 6-, 8-, 10-, 12- and 14-weeks post-challenge. A linear mixed-effects (multilevel) model was fitted between the response (weight) and predictor (diet) by allowing the intercepts and slopes of the time variable (day) to vary to account for tank level correlations and variability in growth trajectories. The model was estimated with the lmer function in the lme4 package of the R language (R Development Core Team, 2008). All treatment effects were based on posterior simulation (n = 2,500) with 95% credible intervals that were interpreted as statistical significant (p = 0.05%) when the interval did not overlap the reference value in question. There were no significant differences in overall growth between dietary treatments at the end of the trial (i.e. after 192 days) (Additional file 2: Figure S1). However, after 92 days of feeding (pre-challenge) fish fed diet CMS2 had higher weights than those fed REF and CMS1 diets.

### PMCV challenge

After the pre-feeding period, two fish from each pre-challenge tank, 18 in total (n = 6 per dietary treatment) were collected, as described below, prior to challenge (0 time-point) and the remaining fish were transferred to challenge tanks (1 m^3^). The fish were unvaccinated and tested for PMCV, ASRV, infectious pancreatic necrosis virus (IPNV) and salmon pancreas disease virus (SPDV) and confirmed as free from CMS, HSMI, IPN and PD. Of the available salmon, a total of 600 fish, pre-challenge average weight 479 ± 13 g for the fish fed with the REF diet; 475 ± 13 g for the fish fed with the CMS1 diet and 502 ± 14 g for the fish fed with the CMS2 diet; were distributed into 12 tanks (4 tanks per dietary treatment with 50 fish/tank). The fish were acclimated for 2 weeks prior to challenge and were fed with the same diets during the acclimation period and throughout the period of the challenge (14 weeks) that they were fed prior to transfer. No previous diseases were described. For challenge, the fish were sedated using Aqui-S at a final concentration of 5 mg/L of isoeugenol, followed by anaesthesia in benzocaine (20%, Benzoak®) using a final concentration of 30 ml/L. All 576 fish were challenged by intramuscular injection of 0.1 ml PMCV inoculum on each side of the fish in the lateral muscle tissue beneath the dorsal fin (0.2 ml in total per fish). Production of the PMCV inoculum was described previously [1]. Briefly, the virus was originally isolated from heart tissue homogenate collected from a clinical outbreak of CMS and filtered through a 0.22-μm filter. Challenge of Atlantic salmon was performed using supernatant of GF-1 cell culture in which the virus was inoculated.

### Sampling

Ten fish from each tank (total of 40 per dietary treatment) were sampled at 6-, 8-, 10-, 12- and 14-weeks post-challenge. Fish were anaesthetized as above and killed by a blow to the head and heart tissue collected for analyses. Liver tissue and a portion of each heart were transferred to 10% buffered formalin for histological analyses. Half of the remaining portions of heart tissue were immediately transferred to RNAlater® (following manufacturers protocol) and later stored at -20^°^C prior to molecular analyses (i.e. 5 samples of heart tissue per tank, 20 per dietary treatment). The remaining portions of heart tissue were frozen in liquid N_2_ and stored at -80oC prior to lipid analyses (five per tank, 20 per treatment).

### Histology

The histological changes in heart were assessed as described in detail previously [1]. Briefly, the inflammatory changes were evaluated separately for atrium and ventricle, and to the extent present, inflammatory scores of the epicardium were also recorded. In addition to atrium and ventricle in the same sample, *bulbus arteriosus* was also included but not assessed for histopathological changes. Samples were fixed in 10% buffered formalin, embedded in paraffin wax and sectioned at 3 - 4 micron and stained with hematoxylin and eosin according to standard methods. All sections were evaluated randomly and without knowledge to which feeding group they belonged (double-blind).

Liver steatosis scores were ranked according to a non-continuous score grade from 0 to 4 (Table 6). Briefly, a score of 0-1 indicated lesions in the cytoplasm involving less than 10% of the hepatocytes and including less than 25% of the area of the individual hepatocytes, and low levels of leukocyte infiltration. A score of 4 indicated lesions in the cytoplasm involving more than 75% of the hepatocytes and including more than 80% of the area of the individual hepatocytes, and severe leukocyte infiltration. The initial “continuous” scores were converted to a fewer number of discrete scores (3 - 6 classes depending on the response). Scores were an ordinal response and a multilevel ordinal regression was used to analyse the effects of feeds on the scores. This was achieved with the ordinal package Sweave le processed by LATEX of the R language (http://www.R-project.org). Each of the scores was used as the response variable in separate analyses that had feed type (REF, CMS1, CMS2) as the fixed effect term. Since multiple fish were examined from each replicate tank, a random effect of tank was added to the model.

**Table 6:** **Scoring system for liver steatosis in individual sections**

Score	Type of changes
0	No pathological findings or negligible number of inflammatory cells present.
0.1-0.9	Increasing number of inflammatory cells per foci and moderate myocyte necrosis per foci.
1	One or a few distinct focal lesions with cardiomyocyte necrosis, and distinct infiltration of leukocytes (1-5 per foci)
1.1-1.9	Increasing number of lesion individually increasing in size per focus
2	Several distinct lesions and increased number of leukocytes
2.1-2.9	Multifocal myonecrosis with increasing number of inflammatory cells
3	Multifocal to confluent lesions and moderate to severe increase in number of leukocytes
3.1-3.9	Increasing number of foci towards diffuse lesions. Inflammation increases as do endothelial hyperplasia.
4	Severe confluent lesions comprising >75% of the tissue and massive leukocyte infiltration

The changes observed by histological examination have been indicated on a visual analog scale (0-4) based on the criteria given above.

### Lipid analyses

Lipid and fatty acid analyses were performed on heart tissue from all sampling points. Three pre-challenge heart samples per dietary treatment were randomly chosen for lipid analysis. Heart tissue from the post-challenge samples was pooled per tank so that heart tissue of five fish became a pool resulting in 4 pools/treatment/sampling point. Total lipid from each pool was extracted according to Folch et al. [51] by homogenization in chloroform/methanol (2:1, v/v) containing 0.1% butylated hydroxytoluent (BHT) and lipid content determined gravimetrically. Total lipid extracts were re-suspended in chloroform/methanol at a concentration of 10 mg lipid/ml and stored at -70^oC^ until analysed. Phospholipid (PL) fractions were prepared from 0.5 mg of total lipid by thin-layer chromatography as described by Bell et al. [52]. FAME were separated and quantified by GLC (Fisons 8160; Carlo Erba, Milan, Italy) using a 60 m × 0.32 mm × 0.25 μm film thickness capillary column (ZB-WAX; Phenomenex, Macclesfield, Cheshire, UK) as also described previously [52]. Tissue and diet lipid class compositions were determined by single-dimension double-development high-performance thin-layer chromatography (HPTLC) and densitometry as described previously [22]. Significance of differences due to diet and time were determined by two-way ANOVA (p < 0.05) using the SPSS 19.0 statistical package (SPSS Inc., Chicago IL, USA).

### Microarray experimental design

Heart tissue, as the main organ affected during a CMS outbreak, was selected for transcriptomic analysis. The choice of appropriate time-points for analysis was based on the description of expression of genes of the immune response after a PMCV infection described by Timmerhaus et al. [10]. Thus samples from fish at pre-challenge, 6-, 8-, and 14- weeks post-challenge (wpc) were analyzed. The pre-challenge sampling point was selected to evaluate changes due to dietary compositions, and 6- and 8-wpc sampling points were selected to evaluate dietary effects on the expression of genes from immune response pathways at the peak of infection. As a plateau phase in the expression of immune related genes at 10- and 12-wpc was described previously [10], the 14-wpc sampling point was selected to evaluate gene expression during the recovery phase of the disease. Within each of these sampling points, the available samples were arranged by individual histoscore and 6 samples per dietary group were randomly selected from the interquartile range (25 – 75%).

### RNA extraction and purification

Heart tissue of the selected samples was homogenized in TRI Reagent (Ambion, Applied Biosystems, Warrington, U.K.) using an Ultra-Turrax homogenizer (Fisher Scientific, Loughborough, U.K.). Total RNA was isolated following manufacturer’s instructions, and RNA quality and quantity assessed by gel electrophoresis and spectrophotometry (NanoDrop ND-1000, Thermo Scientific, Wilmington, U.S.A.), respectively.

### Microarray hybridisations and analysis

The transcriptomic experiment used an Atlantic salmon custom-made oligoarray with 44 k features per array on a four-array-per-slide format, with each feature printed singly (Agilent Technologies UK Ltd., Wokingham, UK). The probes were co-designed by researchers at the Institute of Aquaculture, University of Stirling, U.K. and Nofima (Ås, Norway), and array design is available on request. A dual-labelled experimental design was employed for the microarray hybridizations. Each experimental sample was competitively hybridized against a common pooled-reference sample, which comprised equal amounts of each of the replicates used in the study. This design permits valid statistical comparisons across all treatments to be made. The entire experiment comprised 72 hybridizations; 4 time-points (pre-challenge, 6-, 8- and 14-wpc) × 3 diets (REF, CMS1 and CMS2) × 6 biological replicates.

Indirect labelling methodology was employed in preparing the microarray targets. Amplified antisense RNA (aRNA) was produced from each RNA sample using the TargetAmp™ 1-RoundAminoallyl-aRNA Amplification Kit (Ambion, Applied Biosystems, Warrington, UK), following the manufacturer’s methodology, followed by Cy3 or Cy5 fluor incorporation through a dye-coupling reaction. Briefly, 250 ng of total RNA per sample were amplified and column-purified according to manufacturer’s instructions. Resultant aRNA was quantified and quality assessed as above. Subsequently, Cy dye suspensions (Cy3 & Cy5) in sufficient quantity for all labelling reactions were prepared by adding 38 μL high purity dimethyl sulphoxide (Stratagene, Agilent Technologies UK Ltd.) to each tube of Cy dye (PA23001 or PA25001, GE Healthcare, Chalfont St. Giles, UK). To attach the Cy dyes, 3 μg each aRNA sample was suspended in 10 μL nuclease-free H_2_O and heated to 70°C for 2 min. When cooled to room temperature, 3 μL of coupling buffer (0.5 M NaHCO_3_; pH 9.2) and 2 μL of Cy3 dye suspension stock was added and then incubated for 1 h at 25°C in the dark. To label the common pooled reference sample with Cy5, a scaled-up batch reaction was similarly performed. Unincorporated dye was removed by column purification (Illustra AutoSeq G-50 spin columns; GE Healthcare). Dye incorporation and aRNA yield were quantified by spectrophotometry (NanoDrop) and further quality controlled by separating 0.4 μL of the sample on a thin mini-agarose gel and visualizing products on a fluorescence scanner (Typhoon Trio, GE Healthcare).

Hybridization of a total of 6 slides (24 arrays) was performed in a single day, with sample order semi-randomized, using SureHyb hybridisation chambers in a DNA Microarray Hybridization Oven (Agilent Technologies) as described previously [25]. Scanning was performed at 5 μm resolution using an Axon GenePix 4200AL Scanner (MDS Analytical Technologies, Wokingham, Berkshire, U.K.). Laser power was kept constant (80%) and the “auto PMT” function within the acquisition software (v.4) was enabled to adjust PMT for each channel such that less than 0.1% of features were saturated and that the mean intensity ratio of the Cy3 and Cy5 signals was close to one. Agilent Feature Extraction Software (v 9.5) was used to identify features and extract fluorescence intensity values from the resultant TIF images. The remaining analysis was then performed in the GeneSpring GX version 12 analysis platform (Agilent Technologies). After removing control features and filtration of low quality spots (saturated, non-uniform, population outliers and spots non-significantly different from background), lowess normalization of log2-expression ratios (ER) was performed.

As the primary objective of the present study was to evaluate the effects of functional feeding (i.e. comparison between diets), differentially expressed genes were selected based on expression changes (cut off log2-ER = 0.8, 1.75-fold change, p < 0.05, one-way ANOVA) between the functional feed groups (CMS1 and CMS2) and the fish fed the REF diet, or between the two functional feed groups at each sampling point (pre-challenge-, 6-, 8- and 14-wpc). Annotation of selected genes was performed using STARS software developed at Nofima (Ås, Norway) [53], KEGG analysis, and information from literature and public databases such as Gene Expression Omnibus (GEO).

### Validation of the microarray

Expression of 11 selected genes showing a significant diet × time interaction in the microarray analysis at 2 different time points of the infection and related to relevant immune-related pathways was studied by reverse transcription quantitative real-time PCR (qPCR) as previously described [25]. The qPCR primer sequences, annealing temperature (Tm) and size of amplicon are given in Additional file 3: Table S2. The sequences were obtained either by literature searches or designed from EST sequences corresponding to microarray clones or candidate genes of interest using Primer3 software (http://biotools.umassmed.edu/bioapps/primer3_www.cgi). *Cofilin-2* and *elf-1α* were used as reference genes as they were sufficiently stable across treatments for normalization and had been identified in previous salmon cDNA microarray and qPCR studies as suitable reference genes on the basis of constant expression between different feeds and time points [44, 54]. Data were analysed using the relative expression software tool (REST 2009, http://www.gene-quantification.info/), which employs a pair-wise fixed reallocation randomization test (10,000 randomizations) with efficiency correction, to determine the statistical significance of expression ratios (or gene expression fold-changes) between two treatments [55]. There was good agreement between microarray results and RT-qPCR quantification, with most of the comparisons showing identical regulation and similar fold differences (Additional file 4: Table S3). Analysis indicated that the gene expression data obtained using the oligoarray were reliable and robust, which enabled interpretations to be made with confidence, especially with immune response-related genes.

### Assessment of viral load

Relative quantification of PMCV by qPCR was used to determine differences in the viral load between the dietary treatments at the two critical points in the time-course of the infection (6- and 8- weeks post-challenge) when differential expression of genes related with the inflammatory process was greatest between fish fed the REF diet and fish fed the functional feeds. The qPCR primer sequences (Additional file 3: Table S2) previously described by Timmerhaus et al. [11] were employed following the same procedure and from the same RNA samples used for the validation of the microarray (as described above).

### Ethical statement

All experimentation performed by the Institute of Aquaculture (IoA) is subjected to a thorough Ethical Review Process prior to any work being approved. This involves all projects, irrespective of where they are carried out, to be submitted to the IoA Ethical Committee for approval using detailed Ethical Approval forms that require all aspects of the experimentation to be described including conditions for the human experimenters as well as animal subjects. This procedure ensures all ethical issues are addressed before an experiment can be initiated. The present research was assessed by the IoA Ethical Review Committee and passed the Ethical Review Process of the University of Stirling.

### Availability of supporting data

The data set supporting the results of this article is included within the article (Additional file 1: Table S1) and microarray design is available in ArrayExpress repository, A-MEXP-2400.

## Electronic supplementary material

Additional file 1: Table S1: Gene expression pre-challenge, 6-, 8- and 14- weeks post challenge. Metabolic genes selected by significance (one-way ANOVA at each time point) and expression differences between the functional feeds (CMS1 and CMS2) and reference diet (REF) or between CMS dietary groups. At cut-off log2-ER = 0.8 (1.75-fold). Red/orange colour intensity was used to indicate higher expression and green/blue colour intensity was used to indicate lower expression. (XLSX 292 KB)

Additional file 2: Figure S1: Growth performance over the course of the entire trial, before and after the viral challenge. Error bars denote approximate 95% confidence limits. (PDF 96 KB)

Additional file 3: Table S2: Primers used for the RT-qPCR. (XLSX 15 KB)

Additional file 4: Table S3: RT-qPCR validation of microarray results. Values represent the expression ratios for the selected genes between the three dietary groups at 6- and 8-weeks post-infection with PMCV obtained by microarray analysis or RTqPCR. (XLSX 12 KB)
