# Supplementary figures and images for: Effects of functional feeds on the lipid composition, transcriptomic responses and pathology in heart of Atlantic salmon (Salmo salar L.) before and after experimental challenge with Piscine Myocarditis Virus (PMCV)
Source: BMC Genomics. 2014 Jun 11;15(1):462. doi: 10.1186/1471-2164-15-462 (PMC4079957; doi:10.1186/1471-2164-15-462)

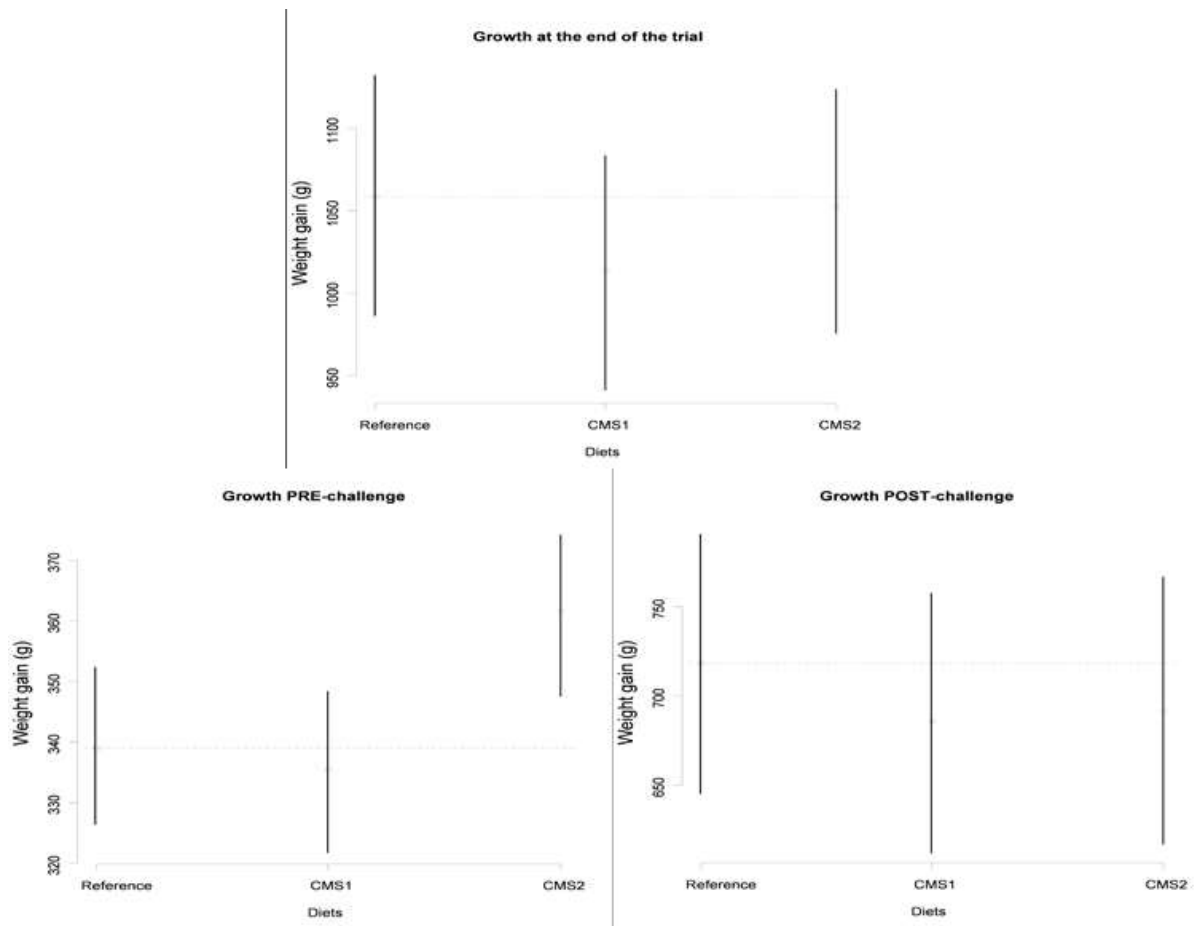

Supplement: Supplementary file 2 — Additional file 2: Figure S1: Growth performance over the course of the entire trial, before and after the viral challenge. Error bars denote approximate 95% confidence limits. (PDF 96 KB) [file 12864_2014_6183_MOESM2_ESM.pdf]
